# In Silico Analysis Identifies Upregulated lncRNA DLGAP1-AS1 Which Is Correlated to Poor Prognosis and Promotes Cell Proliferation in Glioblastoma

**DOI:** 10.1155/2022/5038124

**Published:** 2022-03-15

**Authors:** Yinxing Huang, Li Liu, Meng Fang, Wangjun Yan

**Affiliations:** ^1^Fuzhong Clinical Medical College of Fujian Medical University, Fuzhou, China; ^2^Department of Neurosurgery, 900th Hospital, Fuzhou, China; ^3^School of Materials Science & Engineering, University of Shanghai for Science and Technology, Shanghai, China; ^4^Department of Musculoskeletal Surgery, Fudan University Shanghai Cancer Center, Shanghai, China; ^5^Department of Oncology, Shanghai Medical College, Fudan University, Shanghai, China

## Abstract

Long noncoding RNAs have been reported to regulate the tumorigenesis, growth, and metastasis of glioblastomas. In this study, we identified 1623 differently expressed mRNAs and 38 lncRNAs utilizing the CGGA and TCGA databases. Among these mRNAs and lncRNAs, we focused on DLGAP1-AS1 in this study. The results demonstrated that DLGAP1-AS1 was higher in WHO IV glioma than in WHO II and WHO III gliomas, higher in WHO III glioma than in WHO II glioma samples, higher in IDH1 wildtype glioma than in IDH1-mutant glioma samples, and higher in 1p/19q noncodeletion glioma than in 1p/19q codeletion glioma samples. Moreover, we observed that higher expression levels of DLGAP1-AS1 were correlated to shorter OS time in both low-grade and high-grade gliomas. Next, we evaluated the function of DLGAP1-AS1 in GBM using in vivo experiments. The data revealed that DLGAP1-AS1 knockdown greatly hindered U87 cell and U251 cell proliferation. Using coexpression network analysis, we identified that ATG4A was a potential downstream target of DLGAP1-AS1. The further analysis showed that ATG4B was significantly upregulated and correlated to shorter OS time in gliomas using both the CGGA and TCGA databases. Finally, we showed that ablated ATG4B greatly hindered GBM cell proliferation. Our conclusion suggested that DLGAP1-AS1 may be a potential prognosis biomarker and facilitated the occurrence and development of glioma via ATG4A in GBM.

## 1. Introduction

The mechanisms of glioblastoma multiforme are not yet being uncovered clearly. Due to high aggressiveness of GBM, the patients' survival time was extremely short [1]. The incidence rate of GBM in the USA is approximately 4.43 among 100,000 peoples [1]. GBM is the widespread malignant neoplasm in the adults' brain [2]. Emerging efforts were paid to identify novel therapy methods for GBM. For example, glioma-specific cytotoxic T lymphocyte (CTL) has been revealed as a potential therapy target against glioma [3]. EGFRvIII is amplified among 20–25% of GBM patients, which was a key mechanism underlying EGFR inhibitor resistance [4, 5]. In addition, effective DNA repair and antiapoptosis were related to chemotherapy and radiotherapy resistance in GBM [6]. Understanding the mechanism of the pathological basis of glioblastoma on the molecular level could provide new therapy strategies.

Long noncoding RNAs (lncRNAs) are an important modulator in a variety of biological processes [7, 8]. lncRNAs not only modulate the expression of genes in the form of a competitive endogenous RNA but also modulate epigenetics via binding to its associated proteins [9, 10]. More and more researches proved that lncRNAs exerted crucial roles in cell proliferation, apoptosis, and prognosis. For instance, PlncRNA-1 facilitates cell proliferation of prostate carcinoma and induces the epithelial-mesenchymal transition. XIST mediated cell proliferation of pancreatic carcinoma via modulating miR-133a/EGFR [11]. E2F accelerated cell proliferation via enhance lncRNA (EPEL). In gliomas, lncRNA CASC2 was displayed as an inhibitor of cell growth through negatively modulating miR-21, whereas lncRNA CRNDE induced cell growth and invasion of glioma via modulating mTOR signaling [12]. Ablating lncRNA HOTAIR could impede glioma cell malignant biological progression via regulating miR-326 [13]. Qin et al. found that lncRNA MEG3 inhibited glioma cell proliferation and migration via exhibiting as a competing endogenous RNAs (ceRNA) of miR-19a [14]. It is confirmed by Xiao et al. that lncRNA TP73-AS1 was displayed as a miR-124-dependent iASPP regulatory inhibitor to hinder the growth and metastasis of glioma [15]. lncRNA PVT1 boosted glioma tumorigenesis and development by modulating the miR-128-3p/GREM1 axis and BMP signaling pathway [16]. lncRNA H19 mediated the promotion of glioma angiogenesis via the miR-138/HIF-1*α*/VEGF axis [17]. lncRNA LINC00115 activated by TGF-*β* is a key modulator of the tumorigenicity of GSCs [18]. Through targeting the miR-338-3p/PKM2 axis, lncRNA LINC00689 enhances glioma cell growth, metastasis, and glycolysis [19]. Current studies had demonstrated that XIST expression had a relationship with human GSCs.

We firstly analyzed DLGAP1-AS1 expression in normal tissues of human glioma. Our results demonstrated that DLGAP1-AS1 was heightened in the tissues of human glioma. In this paper, we present a novel molecular mechanism by which DLGAP1-AS1 induced cell proliferation and metastasis of human glioma. Our data showed that DLGAP1-AS1 accelerated glioma cell migration and invasion via ATG4A.

## 2. Methods

### 2.1. Cell Culture

U87MG and U251 (human GBM cells, ATCC, USA) and HEK-293T cells were maintained in DMEM with 10% FCS and 50 mg/mL penicillin/streptomycin as previously reported.

### 2.2. Bioinformatics Analysis

We obtained the expression data from the CGGA database (http://www.cgga.org.cn/). To explore the main biochemical metabolic pathways involved in genes, we conducted WebGestalt database (http://www.webgestalt.org) for KEGG enrichment analysis of genes.

### 2.3. Cell Transfection

Overexpression plasmids DLGAP1-AS1 and/or ATG4A or siRNAs specific for DLGAP1-AS1 or ATG4A were transfected into U87 and U251 cells. DLGAP1-AS1 or ATG4A cDNA digested by BamHI/EcoRI sites was cloned into pcDNA3.1 construct to obtain DLGAP1-AS1 or ATG4A overexpression cassettes. siRNAs targeting DLGAP1-AS1 or ATG4A were synthesized by GenePharma. Table S1 listed siRNA sequences respective for DLGAP1-AS1 and ATG4A. A density of 3 × 10^5^ GBM cells in each well was plated in 6-well plates. Cells were transfected with 50 nM of siRNAs by Lipofectamine 2000 (Invitrogen, USA). We harvested transfected cells after 48 h transfection and conducted RT-qPCR analysis. Stable and positive cell lines were obtained in the presence of G418 selection (Invitrogen, USA).

### 2.4. RT-qPCR

TRIzol was utilized to harvest whole RNA from cells (15596-026, Invitrogen). RNA was then transcribed into cDNA as kit protocol depicted (K1621, Fermentas). All primer sequences (Table S1), including lncRNA DLGAP1-AS1, ATG4A, ERK, Bax, Bcl-2, survivin, MMP-9, and *β*-actin, were ordered from Shanghai Genechem Co., Ltd. RT-qPCR (Takara, China) used for quantification of relative gene mRNA expression was conducted on ABI 7500 (ABI, USA).

### 2.5. CCK-8 Methods

CCK-8 assay and cell counting methods were taken to detect cell proliferation of GBM. Taken briefly, we digested cells with transfection at 48 h posttransfection and then plated in a 96-well plate in the number of 1 × 10^5^ cells in per well. At the following day, in per well, we added 10 *μ*L of CCK-8 into cells and incubated under 37°C for 4 h. After that, DMEM was discarded, and 100 *μ*L of DMSO was added at room temperature and incubated for 10 min. The absorbance at 490 nm wavelength was detected.

### 2.6. Statistical Analysis

SPSS 16.0 software (SPSS Inc., USA) was taken to analyze the data. We utilized Student's *t*-test or one-way analysis of variance (ANOVA) to compare the difference between groups. The Kaplan-Meier method and the log-rank test were conducted to plot OS curves. Correlations existing in different groups were evaluated by the Pearson correlation coefficient analysis. *P* < 0.05 was regarded as statistical significance.

## 3. Results

### 3.1. Identification of DEmRNA and DElncRNA in Glioma

**Figure 1:**
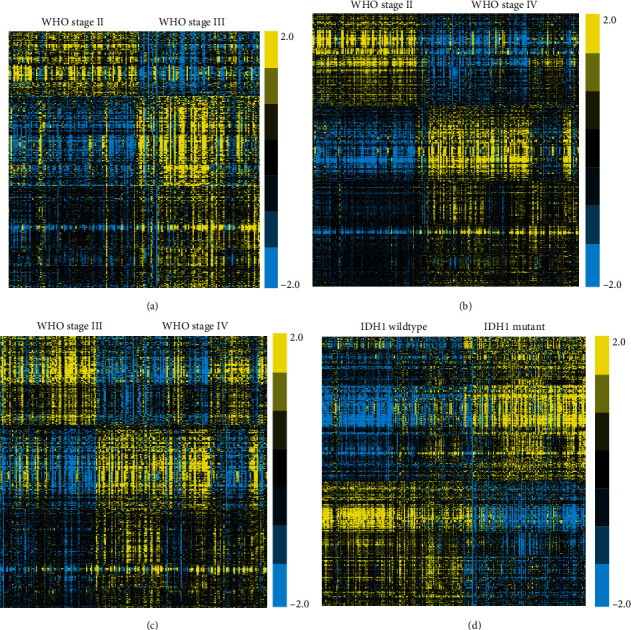
Identification of DEmRNA and DElncRNA in glioma. (a) Heatmap analysis showed DEGs between stages WHO II and III. (b) Heatmap analysis showed DEGs between stages WHO II and IV. (c) Heatmap analysis showed DEGs between stages WHO III and IV. (d) Heatmap analysis showed DEGs between stages IDH1-mutant and IDH1 wildtype gliomas.

To determine the differentially expressed mRNAs (DEmRNAs) and lncRNAs (DElncRNAs) among different stages of human gliomas, we executed expression analysis of public gene expression data from the World Health Organization (WHO) II-IV neoplasms. We obtained expression profiles of lncRNAs from RNA-seq data referring to their Refseq annotation. Then, the DEmRNA and DElncRNA between stages WHO II and III, between stages WHO II and IV, and between stages WHO III and IV were identified. As shown in Figure 1, 2345 genes were found to be upregulated and 802 genes were found to be downregulated in WHO III compared to WHO II samples. 3961 genes were found to be upregulated and 2053 genes were found to be downregulated in WHO IV compared to WHO II samples (Figure 1(a)). 2097 genes were found to be upregulated and 1028 genes were found to be downregulated in WHO IV compared to WHO III samples (Figure 1(b)). Furthermore, we also identified differently expressed genes between IDH1-mutant and IDH1 wildtype gliomas, which is a crucial regulator in glioma progression (Figure 1(c)). After analysis, 5651 genes were differentially expressed between IDH1-mutant and IDH1 wildtype samples. Our data show that among these lncRNAs, 2608 gene expressions were increased and 3043 gene expressions were decreased in IDH1 mutant compared to IDH1 wildtype (Figure 1(d)).

**Figure 2:**
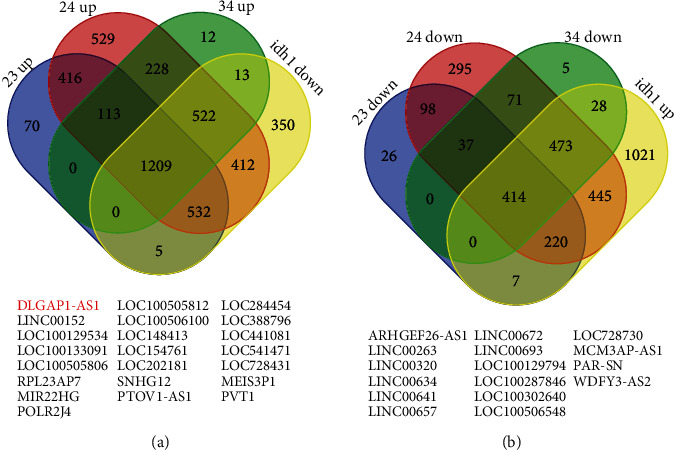
Identification of common DEmRNAs and DElncRNAs in glioma. (a) 1209 genes were identified to be upregulated in advanced stage gliomas and be downregulated in IDH1-mutant gliomas. (b) 414 genes were identified to be downregulated in advanced stage gliomas and be upregulated in IDH1-mutant gliomas.

In order to uncover the key regulators in the progression of gliomas, we conducted further analysis. Finally, 1209 genes were identified to be upregulated in advanced stage gliomas and be downregulated in IDH1-mutant gliomas, indicating that these genes may act as oncogenes in promoting glioma progression (Figure 2(a)). Also, 414 genes were identified to be downregulated in advanced stage gliomas and be upregulated in IDH1-mutant gliomas, indicating that these genes may act as tumor suppressors in inhibiting glioma progression (Figure 2(a)).

Very interestingly, we observed that lncRNAs may also have a crucial role in gliomas. Among these different genes, we identified 22 upregulated lncRNAs (Figure 2(a)), containing DLGAP1-AS1, LINC00152, LOC100129534, LOC100133091, LOC100505806, RPL23AP7, MIR22HG, POLR2J4, LOC100505812, LOC100506100, LOC148413, LOC154761, LOC202181, SNHG12, PTOV1-AS1, LOC284454, LOC388796, LOC441081, LOC541471, LOC728431, MEIS3P1, and PVT1, and 16 downregulated lncRNAs (Figure 2(b)), including ARHGEF26-AS1, LINC00263, LINC00320, LINC00634, LINC00641, LINC00657, LINC00672, LINC00693, LOC100129794, LOC100287846, LOC100302640, LOC100506548, LOC728730, MCM3AP-AS1, PAR-SN, and WDFY3-AS2. Several lncRNAs, such as SNHG12, PVT1, and LINC00672, were shown to function crucially in carcinomas. Among these lncRNAs, DLGAP1-AS1 was one of most attracting differentially expressed lncRNAs and selected for further study because this lncRNA is the most significantly upregulated lncRNA.

### 3.2. Bioinformatics Analysis of DEmRNA in Glioma

**Figure 3:**
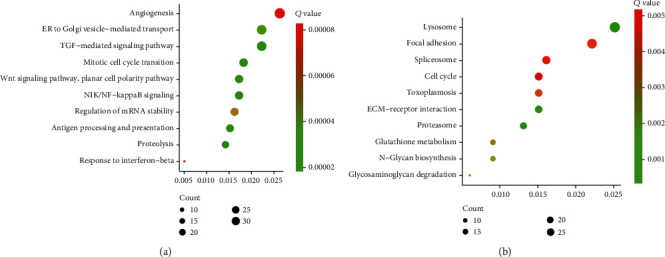
GO and KEGG enrichment analyses of DEmRNAs. (a, b) GO (a) and KEGG (b) enrichment analyses of DEmRNAs.

Then, we performed bioinformatics analysis of DEmRNA in glioma. As presented in the figure, GO analysis showed that DEmRNAs were related to regulate the Wnt signaling pathway, mitotic cell cycle transition, TGF-mediated signaling pathway, ER to Golgi vesicle-mediated transport, and angiogenesis (Figure 3(a)). The KEGG pathway analysis showed that DEmRNAs were related to regulate ECM-receptor interaction, toxoplasmosis, cell cycle, spliceosome, focal adhesion, and lysosome (Figure 3(b)).

### 3.3. DLGAP1-AS1 Was Upregulated in GBM with Poor Prognosis

**Figure 4:**
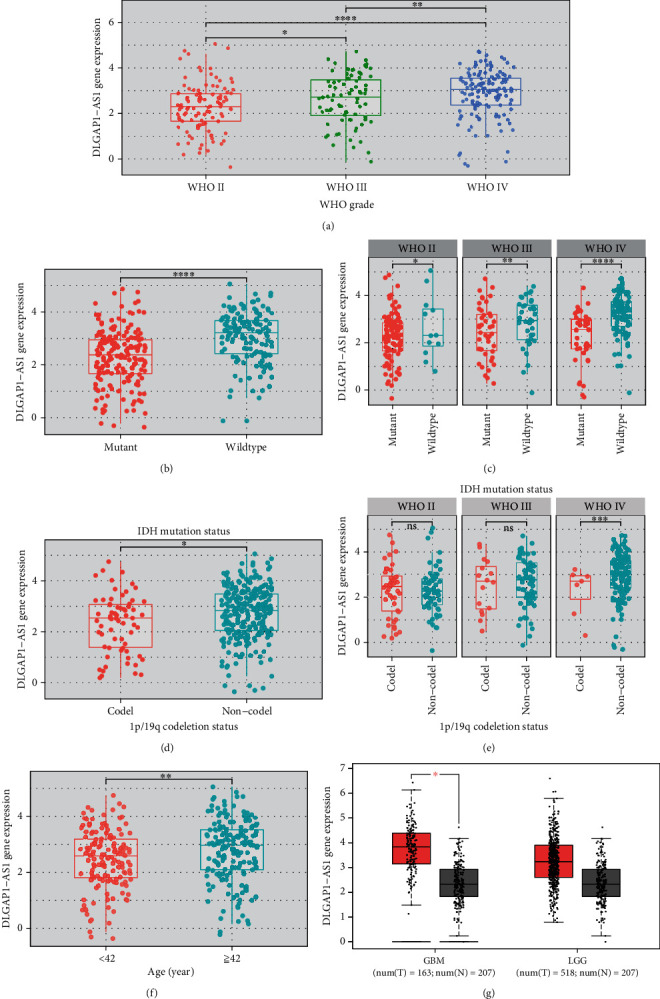
DLGAP1-AS1 was upregulated in GBM. (a) CGGA database analysis showed that DLGAP1-AS1 was differently expressed in WHO II, WHO III, and WHO IV gliomas. (b) CGGA database analysis showed that DLGAP1-AS1 was higher in IDH1 wildtype glioma than in IDH1-mutant glioma samples. (c) CGGA database analysis showed that DLGAP1-AS1 was higher in IDH1 wildtype tumor than in IDH1-mutant tumor samples in WHO II, WHO III, and WHO IV patients. (d) CGGA database analysis showed that DLGAP1-AS1 was higher in 1p/19q noncodeletion glioma than in 1p/19q codeletion glioma samples. (e) CGGA database analysis showed that DLGAP1-AS1 was dysregulated in 1p/19q noncodeletion samples highly expressed DLGAP1-AS1 in WHO II, WHO III, and WHO IV patients. (f) CGGA database analysis showed that DLGAP1-AS1 was higher in glioma patients older than 42 than glioma samples younger than 42 years old. (g) TCGA database analysis showed that GBM and LGG tissues highly expressed DLGAP1-AS1 compared to normal tissues.

We next validated the expression of DLGAP1-AS1 utilizing the TCGA and CGGA cohorts, in view of WHO classification of CNS neoplasms. DLGAP1-AS1 was higher in WHO IV glioma than in WHO II and WHO III gliomas (Figure 4(a)), higher in WHO III glioma than in WHO II glioma samples (Figure 4(a)), higher in IDH1 wildtype glioma than in IDH1-mutant glioma samples (Figure 4(b)), higher in 1p/19q noncodeletion glioma than in 1p/19q codeletion glioma samples (Figure 4(d)), and higher in glioma patients older than 42 than glioma samples younger than 42 years old (Figure 4(f)). By taking into account the clinical stages, we found that DLGAP1-AS1 was higher in IDH1 wildtype tumor than in IDH1-mutant tumor samples in WHO II, WHO III, and WHO IV patients (Figure 4(c)). Compared to 1p/19q codeletion samples, 1p/19q noncodeletion samples highly expressed DLGAP1-AS1 in only WHO IV patients (Figure 4(e)). In order to confirm these findings, we analyze th TCGA database, and the results showed that GBM and LGG tissues highly expressed DLGAP1-AS1 compared to normal tissues (Figure 4(g)). It is found that higher expression of DLGAP1-AS1 was in GBM than in LGG samples (Figure 4(g)). All in all, our data implied that DLGAP1-AS1 level was raised in GBM and was a prospective biomarker for diagnosis.

### 3.4. Highly Expressed DLGAP1-AS1 Exhibited an Association with Poor Survival in Patients with Glioma

**Figure 5:**
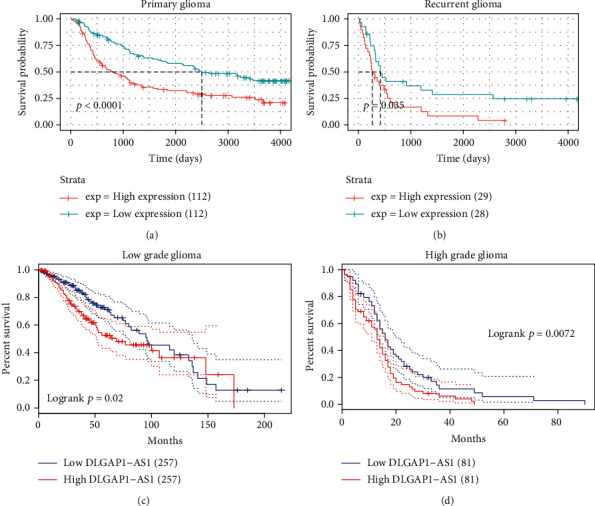
Highly expressed DLGAP1-AS1 exhibited an association with poor survival in patients with glioma. (a, b) Higher expression levels of DLGAP1-AS1 were correlated to shorter OS time in primary and recurrent gliomas. (c, d) Higher expression levels of DLGAP1-AS1 were correlated to shorter OS time in low-grade gliomas and glioblastoma.

We carried out the Kaplan-Meier analysis to detect the relationship of DLGAP1-AS1 expression with patient survival. Both CGGA and TCGA were selected for further analysis. High expression or low expression of DLGAP1-AS1 was determined based on the median expression levels. In both primary and recurrent gliomas, compared with the lowly expressed DLGAP1-AS1 group, the OS time of patients in the highly expressed DLGAP1-AS1 group was greatly shorter by analyzing the CGGA database (Figures 5(a) and 5(b)). Similarly, we also observed that higher expression levels of DLGAP1-AS1 were correlated to shorter OS time in low-grade gliomas and glioblastoma (Figures 5(c) and 5(d)).

### 3.5. DLGAP1-AS1 Knockdown Inhibits the Malignant Phenotypes of GBM

**Figure 6:**
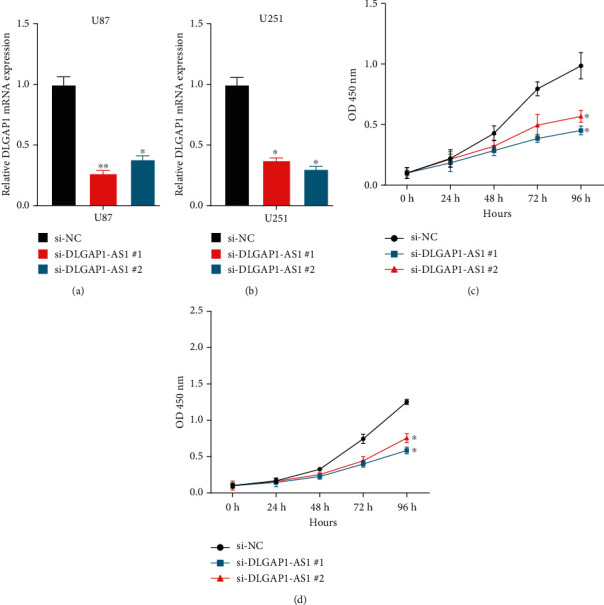
DLGAP1-AS1 knockdown inhibits the malignant phenotypes of GBM. (a, b) DLGAP1-AS1 expression was detected after transfecting with siRNAs in U87 and U251. (c, d) The data revealed that ablated DLGAP1-AS1 greatly hindered U87 cell and U251 cell proliferation.

Here, we evaluated the functions of DLGAP1-AS1 in GBM. Two siRNAs targeting DLGAP1-AS1 were transfected into GMB cell lines. Scrambled siRNA (si-Ctrl) was used as a control (Figures 6(a) and 6(b)). We conducted qRT-PCR to measure the reduction efficiency of DLGAP1-AS1 (Figures 6(a) and 6(b)). Next, we performed CCK-8 assay to detect the effect of DLGAP1-AS1 knockdown on GBM cell proliferation. The data revealed that DLGAP1-AS1 was inhibited largely and ablated DLGAP1-AS1 greatly hindered U87 cell and U251 cell proliferation in patients (Figures 6(c) and 6(d)).

### 3.6. Bioinformatics Analysis Revealed ATG4A Was a Direct Target of DLGAP1-AS1

**Figure 7:**
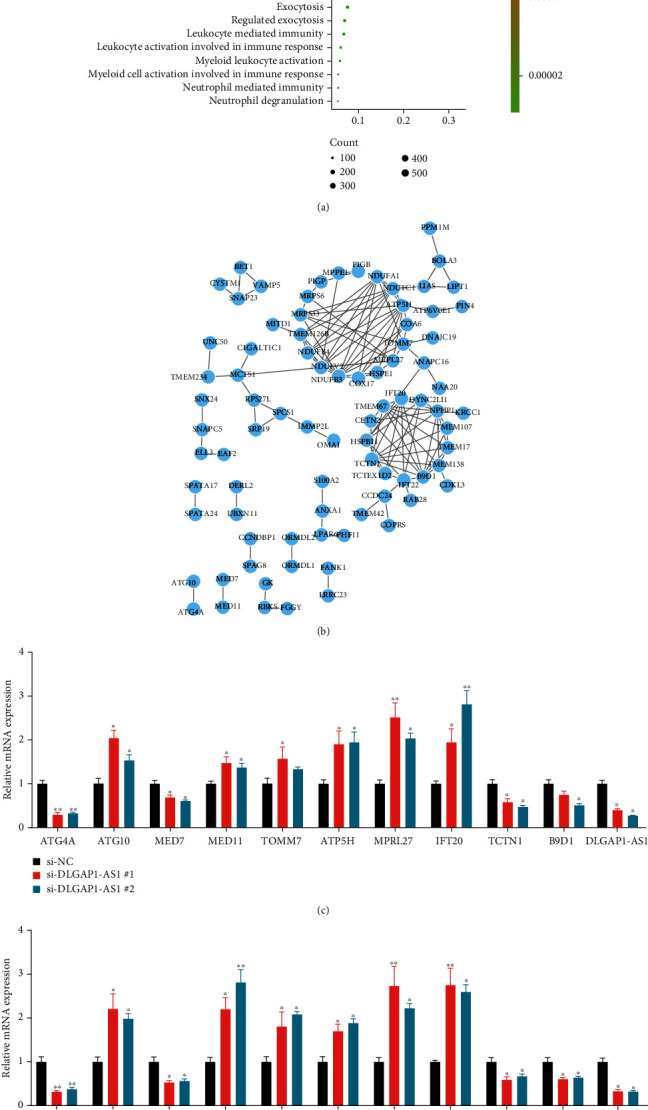
Bioinformatics analysis revealed that ATG4A was a direct target of DLGAP1-AS1. (a) Bioinformatics analysis of DLGAP1-AS1 in gliomas. (b) Construction of PPI networks by using DLGAP1-AS1 targets. (c, d) The expressions of 10 coexpressed genes were detected after knockdown of DLGAP1-AS1 in U87 and U251 cells.

To understand the mechanism of DLGAP1-AS1 in regulating glioma progression, we performed pathway enrichment analysis using the top 100 coexpressed genes. Our results indicated that DLGAP1-AS1 was related to regulate multiple immune-related pathways, such as neutrophil-mediated immunity, myeloid cell activation involved in immune response, myeloid leukocyte activation, leukocyte activation involved in immune response, and leukocyte-mediated immunity (Figure 7(a)).

Also, the protein-protein interaction network was constructed to identify hub targets of DLGAP1-AS1 in gliomas. Of note, among these coexpressed genes, ATG4A was the only one associated with the autophagy signaling pathway which exhibited a high association with the pathogenesis of GBM (Figure 7(b)).

Next, we detected the expression of 10 coexpressed genes after knockdown of DLGAP1-AS1 in U87 and U251 cells, including ATG4A, ATG10, MED7, MED11, TOMM7, ATP5H, MPRL27, IFT20, TCTN1, and B9D1. The results showed that DLGAP1-AS1 silencing remarkably suppressed ATG4A expression in GBM cells (Figures 7(c) and 7(d)).

### 3.7. ATG4B Was Upregulated and Promoted Cell Proliferation of GBM Cells

**Figure 8:**
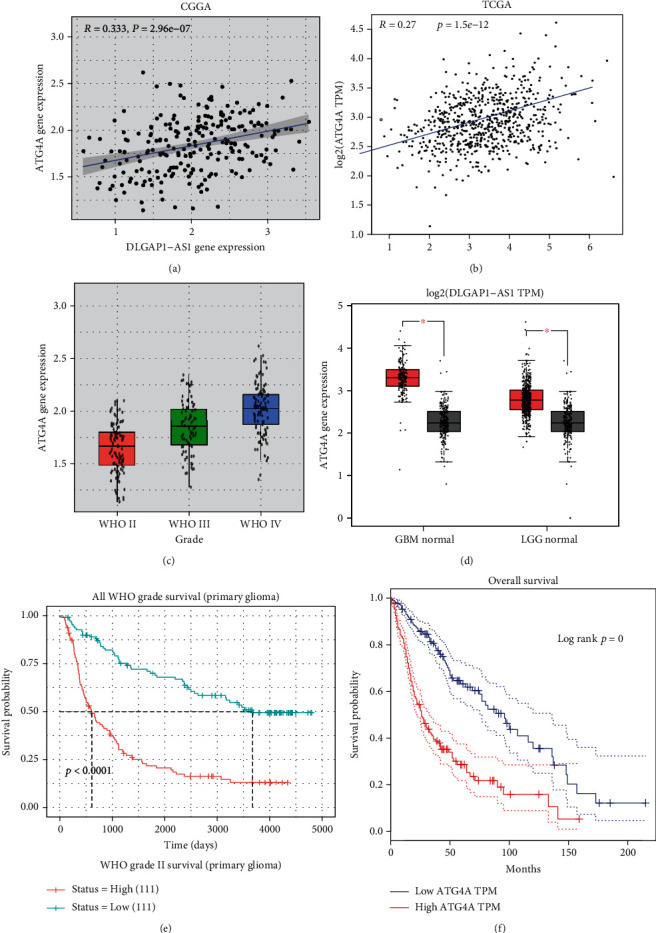
ATG4B was upregulated and promoted cell proliferation of GBM cells. (a, b) DLGAP1-AS1 expression presented a positive correlation with ATG4A using both the TCGA database and CGGA database. (c) CGGA dataset analysis showed that ATG4A expression was positively correlated to advanced stage of gliomas. (d) TCGA databases analysis showed that ATG4B was significantly upregulated in GBM and LGG samples compared to normal samples. (e, f) The Kaplan-Meier analysis showed that higher expression levels of ATG4B were correlated to shorter OS time in gliomas using both the CGGA and TCGA databases.

ATG4B is a key regulator of autophagy. However, its expression and functions in GBM remained to be unclear. In this study, we further verified that DLGAP1-AS1 expression presented a positive correlation with ATG4A using both the TCGA database (*r* = 0.333, *P* < 0.001; Figure 8(a)) and CGGA database (*r* = 0.27, *P* < 0.001; Figure 8(b)).

Then, CGGA dataset analysis showed that ATG4A expression was positively correlated to advanced stage of gliomas (Figure 8(c)). And TCGA databases analysis showed that ATG4B was significantly upregulated in GBM and LGG samples compared to normal samples (Figure 8(d)). The Kaplan-Meier analysis showed that higher expression levels of ATG4B were correlated to shorter OS time in gliomas using both the CGGA and TCGA databases (Figures 8(e) and 8(f)).

**Figure 9:**
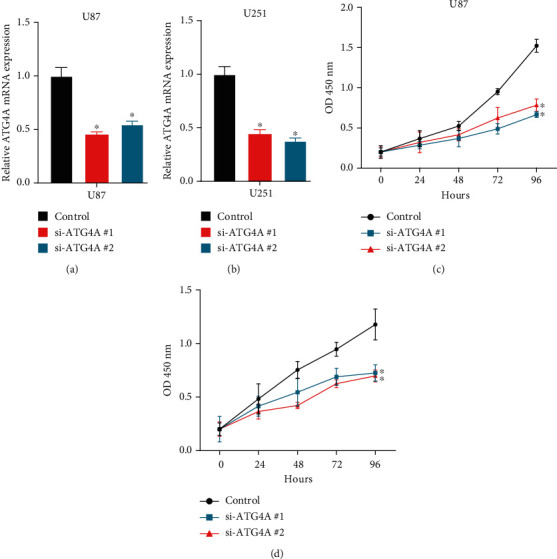
ATG4B knockdown inhibits the malignant phenotypes of GBM. (a, b) ATG4B expression was detected after transfecting with siRNAs in U87 and U251. (c, d) The data revealed that ablated ATG4B greatly hindered U87 cell and U251 cell proliferation. Scramble siRNA (siNC) is the control group. siNC will have no match with any mRNA of the selected organism database and no known miRNA SEED recognition sequence. We have modified the description in the figure legends.

Next, we evaluated the function of ATG4B in GBM. Two siRNAs targeting ATG4B were transfected into GMB cell lines (Figures 9(a) and 9(b)). The data revealed that ablated ATG4B greatly hindered U87 cell and U251 cell proliferation in GBM cells (Figures 9(c) and 9(d)).

## 4. Discussion

Glioma is one of the widely detrimental neoplasms of human brain. In our study, we dug out the different expressed mRNAs and lncRNAs during the progression of glioma. Totally, 1486 mRNAs and 135 lncRNAs were revealed to display a key role in glioma. Bioinformatics analysis showed that these genes were associated with NIK/NF-kappaB signaling, mitotic cell cycle transition, TGF-mediated signaling pathway, proteolysis, antigen processing and presentation, and Wnt signaling pathway, suggesting that the dysregulation of immune response may contribute to the tumorigenesis and progression of glioma. Interestingly, several previous reports had confirmed our findings. For example, most nontumor cells are tumor-associated macrophages (TAMs), which promoted neoplasm cell proliferation, survival, and migration. Our results for the first time showed that DLGAP1-AS1 was overexpressed in glioma and correlated to shorter OS time in both low-grade and high-grade gliomas. Finally, we showed that ablated ATG4B greatly hindered GBM cell proliferation.

Among 38 differently expressed lncRNAs, we focused on DLGAP1-AS1, which had been confirmed as a key regulator of human cancers. DLGAP1-AS1 sequestered miR-486-5p to promote cell proliferation of hepatocellular cancer [20], and DLGAP1-AS1 urged the occurrence of liver cancer and epithelial-mesenchymal transition through the feedback loop of the miR-26a/b-5p/IL-6/JAK2/STAT3 and Wnt/*β*-catenin pathways [21]. Downregulating DLGAP1-antisense RNA 1 attenuated the injury of vascular endothelial cells by activating the phosphatidylinositol 3-kinase/Akt pathway in the rat model of acute limb ischemia [22]. DLGAP1-AS1 stimulates gastric carcinoma invasive behavior via exhibiting as a ceRNA of microRNA-628-5p and increases astrocyte gene 1 expression. Nevertheless, the parts of DLGAP1-AS1 in glioma remained largely unknown. Here, we observed that DLGAP1-AS1 was upregulated in grade 4 compared to grade 3, grade 3 compared to grade 2, and grade 4 compared to grade 1 gliomas. Moreover, the results showed that higher expression levels of DLGAP1-AS1 correlated to shorter OS time in glioma. Our data suggested that DLGAP1-AS1 was a novel biomarker for glioma patients' prognosis. We also explored the potential functions of DLGAP1-AS1 using loss-of-function assays. Our results displayed that the reduction of DLGAP1-AS1 caused glioma cell proliferation inhibition, indicating that this lncRNA acts as an oncogene in glioma. Combining the experimental validation and bioinformatics analysis, we confirmed ATG4A as a downstream target of DLGAP1-AS1. Knockdown of DLGAP1-AS1 suppressed the expression of ATG4A. Using a public database, we revealed that DLGAP1-AS1 expression was positively correlated with ATG4A.

ATG4A is a redox-modulated cysteine protease, known as an autophagy regulator. During autophagy, the C-terminal arginine of ATG8 was removed by ATG4A and then conjugated to phosphatidylethanolamine [23]. In addition to autophagy, some reports have shown the function of ATG4A in tumor initiation. Previous researches revealed that ATG4A probably had a relationship with the stem-like phenotype, drug resistance, and neoplasm metastasis [24, 25]. For example, upregulating ATG4A would promote the transition of osteosarcoma cell epithelial to mesenchymal via the Notch signaling pathway [26]. In our literature, we identified ATG4A as a downstream target of DLGAP1-AS1. In this study, we observed that ATG4A was upregulated in gliomas and correlated to shorter OS time in glioma. Furthermore, we showed that ATG4A knockdown suppressed cell proliferation. Our results showed that targeting DLGAP1-AS1/ATG4A may prove a potential biomarker for glioma treatment.

Several limitations should be noted in this study. First, the data used in this study were extracted from CGGA data. The expression levels of these lncRNAs should be confirmed using clinical samples. In addition, bioinformatics analysis showed that DElncRNAs were related to various signaling, such as angiogenesis. More validation should be performed in the future study. In addition, more loss-of-function assays should be performed to detect the effect of DLGAP1-AS1 on GBM progression, such as migration and the immunofluorescence staining of BrdU and Ki67. Finally, the in vivo assay should be performed, which could strengthen the findings of this study.

In summary, the present study identified differently expressed mRNAs and lncRNAs during the development of glioma. Bioinformatics analysis presented that they exhibited a significant relationship with immune response in glioma. lncRNA DLGAP1-AS1 and ATG4A were overexpressed in the tissues and cells of glioma. At the same time, the regulated links between DLGAP1-AS1 and ATG4A were also explored. Our findings capitulated that DLGAP1-AS1 targeted ATG4A to motivate glioma occurrence and development.

## Data Availability

We obtained the expression data from the CGGA database (http://www.ncbi.nlm.nih.gov/geo). To explore the main biochemical metabolic pathways involved in genes, we conducted the WebGestalt database (http://www.webgestalt.org) for KEGG enrichment analysis of genes.

## Abbreviations

GBM: Glioblastoma
CTL: Cytotoxic T lymphocyte
lncRNAs: Long noncoding RNAs
EPEL: E2F accelerated cell proliferation via enhance lncRNA
ceRNA: Competing endogenous RNAs
DEmRNAs: Differentially expressed mRNAs
DElncRNAs: Differentially expressed lncRNAs
si-Ctrl: Scrambled siRNA
WHO: World Health Organization
TAMs: Tumor-associated macrophages.

## Supplementary Materials

Supplementary Materials Table S1: the sequence of the primers and siRNAs used in this study.
