# Supplementary material for: In Silico Analysis Identifies Upregulated lncRNA DLGAP1-AS1 Which Is Correlated to Poor Prognosis and Promotes Cell Proliferation in Glioblastoma
Source: Comput Math Methods Med. 2022 Mar 15;2022:5038124. doi: 10.1155/2022/5038124 (PMC8941517; doi:10.1155/2022/5038124)
Supplement: Supplementary Materials — Table S1: the sequence of the primers and siRNAs used in this study. [file 5038124.f1.pdf]

primers			
**gene**	**primer label**	**primer sequence**	**primer length**
ATG4A	Forward	TGCTGGTTGGGGATGTATGC	20
	Reverse	GCGTTGGTATTCTTTGGGTTGT	22
ATG10	Forward	AGACCATCAAAGGACTGTTCTGA	23
	Reverse	GGGTAGATGCTCCTAGATGTGAC	23
MED7	Forward	ATCCGCCCTTTGGAAAGTCAG	21
	Reverse	TGGTGGGGTCGGTATTCATTT	21
MED11	Forward	TGGCGAACGAGAGACTACG	19
	Reverse	ACAGTACCTGCATTCTGAAGGAT	23
TOMM7	Forward	CATTCGCTGGGGCTTTATCC	20
	Reverse	TCTGCACCCCTCTTAAATCCC	21
ATP5H	Forward	GCTGGGCGAAAACTTGCTCTA	21
	Reverse	CCAGTCGATAGCTGGTGGATT	21
MPRL27	Forward	CGGACAGCCGTTACATCCTTG	21
	Reverse	CTACCACCCGACTTCTTGGA	20
IFT20	Forward	AGCAGACCATAGAGCTGAAGG	21
	Reverse	AGCACCGATGGCCTGTAGT	19
TCTN1	Forward	TGTTCAGTCCATCGTCATTCAG	22
	Reverse	GCAAAGGCTAAAGTGTCCAGC	21
B9D1	Forward	TGGAGGAGGGGATCTCACAG	20
	Reverse	CCGTAGGGGTTGGTGCTTTT	20
human b-actin	Forward	AAGGTGACAGCAGTCGGTT	19
	Reverse	TGTGTGGACTTGGGAGAGG	19
DLGAP1-AS1	Forward	GGGGCAGGAGTAAAGTGGAC	20
	Reverse	GGGGCAGGAGTAAAGTGGAC	20

siRNAs		
**gene**	**siRNA**	**siRNA sequence**
ATG4A	siRNA-1	CAGATACAGATGAGCTGGTATGGAT
	siRNA-2	CATCCTAAACCTGGATCCTTCAGTT
DLGAP1-AS1	siRNA-1	GGGATGAGAAGAGACTTCCTGGAGA
	siRNA-2	CATGATCTAGCAGGCCAAATCCTAA
NC	siRNA-1	UUCUCCGAACGUGUCACGUTT

Table S1. The sequence of the primers and siRNAs used in this study.
